# Prevalence and awareness regarding diabetes mellitus in rural Tamaka, Kolar

**DOI:** 10.4103/0973-3930.60005

**Published:** 2010

**Authors:** C. Muninarayana, G. Balachandra, S. G. Hiremath, Krishna Iyengar, N. S. Anil

**Affiliations:** Department of Community Medicine, Sri Devaraj Urs Medical College, Tamaka, Kolar, India; 1 Department of Medicine, R. L. Jalappa Hospital and Research Centre, Tamaka, Kolar, India

**Keywords:** Diabetes, hyperglycemia, prevalence

## Abstract

**Background:**

The worldwide prevalence of diabetes mellitus has risen dramatically in the developing countries over the past two decades. Regular screening of adults is essential for early detection and care. There are limited studies on diabetes awareness and prevalence in rural communities. Hence this prevalence and knowledge assessment study was undertaken. Such data are extremely important to plan the public health policies with specific reference to implementation of National Diabetic Control Program.

**Aims:**

To study the prevalence and awareness of diabetes mellitus in rural areas.

**Settings and Design:**

Cross-sectional, household study.

**Materials and Methods:**

A study on adults and elderly age group in Tamaka village was undertaken. Structured questionnaire was used to assess the knowledge of diabetes and capillary blood screening tests done to detect diabetes.

**Statistical Analysis Used:**

SPSS - 11 software.

**Results and Conclusions:**

Ten per cent of the 311 adults screened had hyperglycemia. Half of the interviewed population had some awareness about diabetes and its symptoms. But more than half (75%) of them were not aware of the long term effects of diabetes and diabetic care. The common perception about diet in diabetes was to avoid sweets, rice and fruits and to consume more ragi, millet and wheat chapattis. Diabetes in young adults is common. Relevant knowledge about diabetes is poor in rural population. Hence community level awareness programs have to be organized. Healthcare providers must be aware of community perceptions and practices.

## Introduction

According to the World Health Organization (WHO) report, India today heads the world with over 32 million diabetic patients and this number is projected to increase to 79.4 million by the year 2030.[1] Recent surveys indicate that diabetes now affects a staggering 10-16% of urban population and (5-8%) of rural population in India.[3,4] There is very little data on the level of awareness and prevalence about diabetes in developing countries like India.[5] Such data is important to plan the public health program. This study was taken up to identify, investigate and evaluate knowledge and practice through exploratory and evaluatory research.

## Materials and Methods

A cross-sectional household study on adults in Tamaka village, Kolar was conducted. A structured questionnaire was used to assess the knowledge of diabetes and capillary blood screening tests were done to detect the diabetes. Basic data regarding awareness, knowledge, traditional beliefs, treatment practices and other issues were included in the questionnaire. The data was analyzed using SPSS - 11 software.

## Results

Table 1 a total of 311 adults were interviewed, (54%) were females and (46%) were males. Most of the surveyed population (60%) and diabetic patients (54.8%) are in the age group of 30-45 years this shows Diabetes in young adults is common. About 43.7% of the respondents were illiterates and 56.3% of them were literates; 80% of the surveyed population is sedentary in occupation (teachers, clerk, business skilled workers and home makers), 50.8% of the respondents were aware of the disease diabetes mellitus (and remaining 49.2% of them were unaware of the diabetes); 70% of the illiterate and 43.5% of the literate were not aware of diabetes mellitus.

**Table 1:** Socio-demographic profile of surveyed population

Socio-demographic profile	Non-diabetics (*n* = 280)		Diabetics (*n* = 31)	
		
	No	%	No	%
Age distribution				
<45	168	60.00	17	54.84
46-64	80	28.57	9	29.03
>65	32	11.43	5	16.13
Sex distribution				
Male	121	43.21	22	70.97
Female	159	56.79	9	29.03
Educational status				
Graduate	22	7.86	7	22.58
Matriculate	131	46.79	15	48.39
Illiterate	127	45.36	9	29.03
Occupational status				
Skilled	110	39.29	19	61.29
Unskilled	57	20.36	5	16.13
Professional	7	2.50	2	6.45
House maker	106	37.86	5	16.13

**Figure 1:**
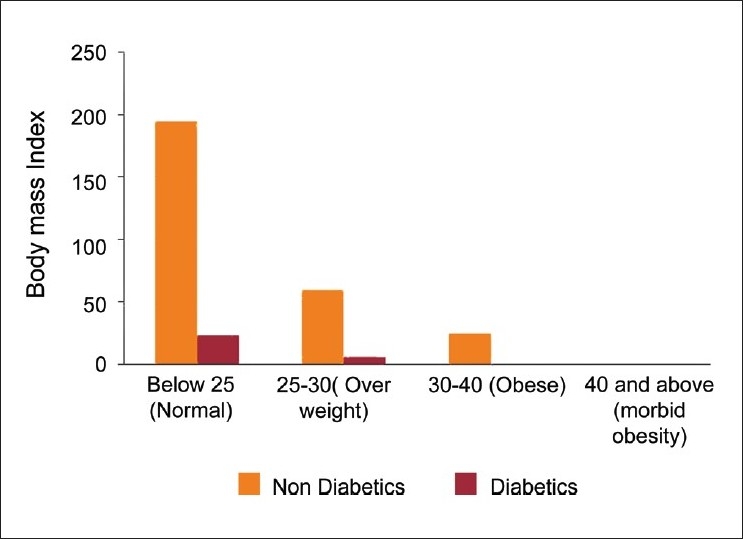
Body mass index among surveyed population

There were 31 cases of diabetes mellitus, 22 (71%) males and nine (29%) are females; prevalence of diabetes mellitus was 10%. Table 2; 70% of participants had normal weight [Figure 1] and only 8.7% of them were obese [Table 3].

**Table 2:** BMI among surveyed population

Body mass index	Non-diabetics (*n* = 280)		Diabetics (*n* = 31)	
		
	No	%	No	%
Below 25 (Normal)	194	69.3	24	77.4
25-30 (Over weight)	60	21.4	06	19.4
30-40 (Obese)	25	8.9	01	3.2
40 and above (Morbid obesity)	1	0.4	0	0

**Table 3:** Perceived causes of diabetes among participants

Causes	Non-diabetics (*n* = 280)		Diabetics (*n* = 31)	
		
	No	%	No	%
Hormonal	30	10.7	10	32.2
Hereditary	45	16.1	14	45.2
Consuming more sweets	77	27.5	9	29.0
Obesity	22	7.8	4	12.9
Others	03	1.1	2	6.4
Don't know	183	65.3	3	9.6

**Figure 2:**
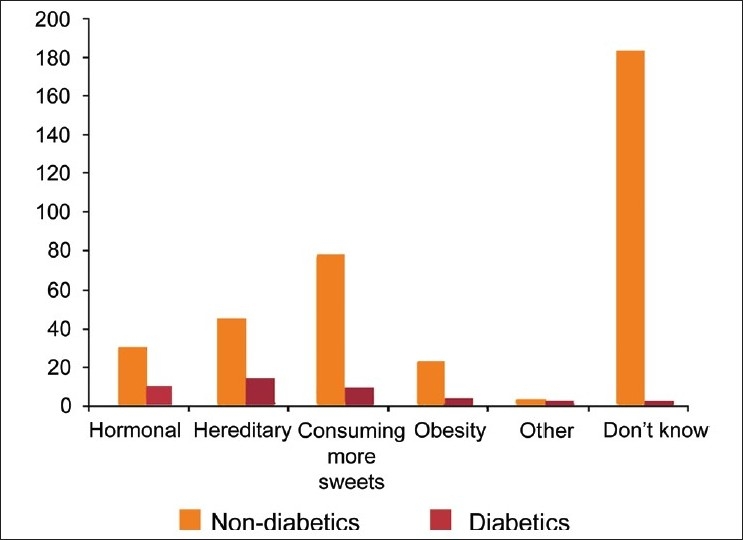
Perceived causes of diabetis among the participants

Only 45% of respondents were aware of the risk factors for diabetes [Figure 2]. Even among diabetics, 16% did not know about the risk factors for diabetes. Knowledge of the role of obesity and physical inactivity in producing diabetes was very low [Table 4].

**Table 4:** Knowledge of diabetes complications among diabetics

Complications	Non-diabetics (*n* = 280)		Diabetics (*n* = 31)	
		
	No	%	No	%
Eye	20	7.1	17	54.8
Heart	16	5.7	14	45.2
Kidney	18	6.4	13	41.9
Joint deformity	14	5.0	11	35.5
Stroke	13	4.6	09	29.0
Diabetic foot	17	6.1	23	74.2
Don't know	205	73.2	08	25.8

**Figure 3:**
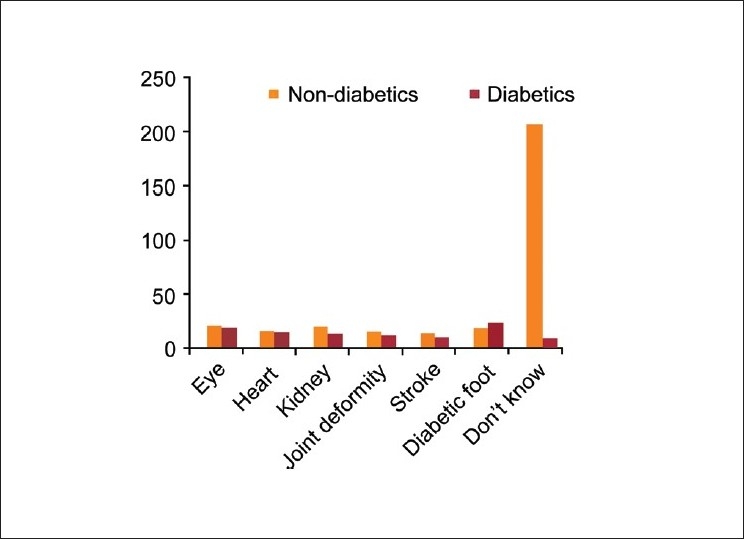
Knowledge of diabetes complications among the diabetics and non-diabetics

Majority (75%) of the participants were not aware of the long term effects of diabetes. The most common complications reported by the non-diabetics participants were eye problems (7.1%) followed by kidney disease (6.4%), foot problems (6.1%) and heart attacks (5.7%) [Figure 3]. All other complications were occasionally mentioned. Even among diabetes subjects only 74.2% were aware that diabetes could produce some complications [Table 5].

**Table 5:** Practices of patients with diabetes mellitus (*n* = 31)

Practices	Diabetics (*n* = 31)	
	
	No	%
Regular medications	31	100
Consult Doctor regularly	3	9.7
Blood glucose monitoring	12	38.7
Regular exercise	17	54.8
Efforts to reduce weight	11	35.5
Cessation of smoking or alcohol habits	21	67.7
Using regular footwear	27	87.1

**Table 6:** Perceived beneficial dietary behavioral changes among diabetics

Perceived beneficial dietary behavioral changes	Diabetics	
	
	No	%
Avoiding sweets	29	93.5
Avoiding fatty foods	27	87.1
Avoiding fasting	21	67.7
Frequent small quantity of feeds	23	74.2
Substitute rice with ragi/wheat	28	90.3

All 31 patients with diabetes were under regular medication but only 54.8% of them exercised regularly; 87.1% of diabetics used footwear regularly. Monitoring of blood sugar was very poor (38.7%). Only (9.7%) of the patients visited doctors on a regular basis [Figure 5].

**Figure 4:**
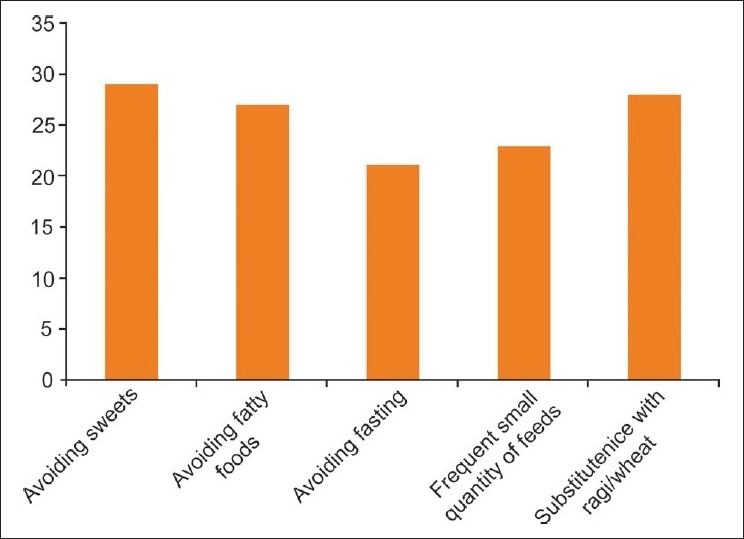
Perceived benificial dietary changes among diabetics

The common perception about diet in diabetics was to avoid sweets (93.5%), rice and fatty foods (87%) and to consume more of ragi millet and wheat chapattis (90%) [Figure 4].

**Figure 5:**
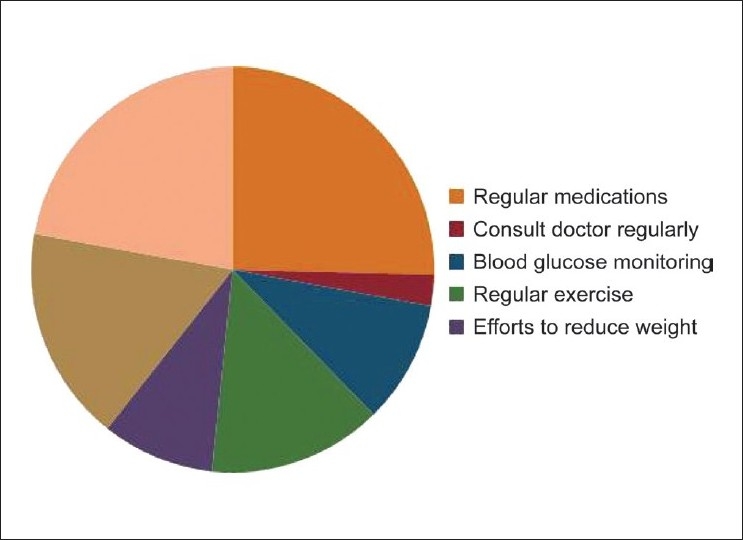
Practices of the patients with diabetes mellitus

## Discussion

The major finding in this study was the lack of awareness of diabetes. Only 50.8% of the participants reported that they knew about a condition called diabetes. A study by Deepa Mohan *et al*. found 75.5% of whole population in Chennai aware of the conditions.[1] Therefore, there is a need to improve the knowledge and awareness about diabetes in the rural as well as in urban areas.

Knowledge about complications of diabetes was even poorer, only 26.8% of non-diabetics and 74.2% of diabetics were aware of the complications. Deepa Mohan *et al*. in Chennai observed that even among self-reported diabetic subjects, knowledge about diabetes including awareness of complications of diabetes was poor.[1] This indicates that majority of the patients have not been taught about diabetes by their physicians. Studies is India and Pakistan show that obesity and over weight problem is less is rural areas compared to Urban areas.[11,12] Similarly our study in rural area showed only 30% of Non diabetic and 22% of the diabetic patients were overweight. This may be due to consuming whole grain meal rather than refined meal and being more physically active and less sedentary than the urban people.[11] Public awareness study by H L Wee in Singapore observed low scores in general knowledge, risk factors of diabetes mellitus but had a good understanding of symptoms and complications of diabetes.[13] This is similar to our study. Gail D Hughes reported that community health workers did not have the requisite knowledge, attitude, and beliefs to make a positive impact on prevention and management of diabetes.[14]

The questions related to risk factors for diabetes revealed that many misconceptions were present and more worrisome was the fact that only 41.2% of non-diabetics and 90.4% diabetics were aware of the risk factors that cause diabetes. As prevention of diabetes is primarily dependent on altering lifestyle and increasing levels of physical activity, changing societal perceptions of health and improving knowledge about the risk factors of diabetes and steps to promote physical activity must receive urgent attention of policy makers and healthcare planners.[5,6]

About 48.4% of the diabetic respondents were not aware of self care in diabetes, study by Kaur and others in Chandigarh observed that 63.3% of them were poor in practicing foot care through regular washing, monitoring of blood sugar was infrequent (46.7%).[2,8] It is likely that the results of the study represent only the tip of the iceberg, in-depth community based studies has to be undertaken to assess the awareness, about diabetes. Community level awareness programs need to be launched to increase awareness.

This study reveals that knowledge regarding diabetes is very poor in rural areas. This emphasizes the need for carrying the right messages regarding diabetes right down to the masses and also extending diabetes education activities to rural areas as well where the prevalence rates of diabetes have already begun to rise.[9] In conclusion, this study reflects the poor knowledge and awareness about diabetes in rural India. This emphasizes the need for increasing diabetes awareness activities in the form of mass campaigns in both urban and rural areas of India.
